# Entropy Generation and Statistical Analysis of MHD Hybrid Nanofluid Unsteady Squeezing Flow between Two Parallel Rotating Plates with Activation Energy

**DOI:** 10.3390/nano12142381

**Published:** 2022-07-12

**Authors:** Nimer Murshid, Hasan Mulki, Mahmoud Abu-Samha, Wahib Owhaib, S. Suresh Kumar Raju, Chakravarthula S. K. Raju, Macherla JayachandraBabu, Raad Z. Homod, Wael Al-Kouz

**Affiliations:** 1 College of Engineering and Technology, American University of the Middle East, Kuwait; mahmoud.abusamha@aum.edu.kw; 2 Mechanical and Maintenance Engineering Department, German Jordanian University, Amman 11180, Jordan; wahib.owhaib@gju.edu.jo; 3 Department of Mathematics and Statistics, College of Science, King Faisal University, P.O. Box 400, Al-Ahsa 31982, Saudi Arabia; ssurapuraju@kfu.edu.sa; 4 Department of Mathematics, GITAM School of Science, GITAM Deemed to be University, Bangalore 562163, India; rchakrav@gitam.edu; 5 Department of Mathematics, S.V.A Government College, Srikalahasti 517644, India; jayamacharla@gmail.com; 6 Department of Oil and Gas Engineering, Basrah University for Oil and Gas, Basrah 61004, Iraq; raadahmood@yahoo.com

**Keywords:** squeezing flow, parallel plates, hybrid nanofluid, activation energy, correlation coefficient, entropy generation, hybrid nanomaterials

## Abstract

Squeezing flow is a flow where the material is squeezed out or disfigured within two parallel plates. Such flow is beneficial in various fields, for instance, in welding engineering and rheometry. The current study investigates the squeezing flow of a hybrid nanofluid (propylene glycol–water mixture combined with paraffin wax–sand) between two parallel plates with activation energy and entropy generation. The governing equations are converted into ordinary differential equations using appropriate similarity transformations. The shooting strategy (combined with Runge–Kutta fourth order method) is applied to solve these transformed equations. The results of the conducted parametric study are explained and revealed in graphs. This study uses a statistical tool (correlation coefficient) to illustrate the impact of the relevant parameters on the engineering parameters of interest, such as the surface friction factor at both plates. This study concludes that the squeezing number intensifies the velocity profiles, and the rotating parameter decreases the fluid velocity. In addition, the magnetic field, rotation parameter, and nanoparticle volumetric parameter have a strong negative relationship with the friction factor at the lower plate. Furthermore, heat source has a strong negative relationship with heat transfer rate near the lower plate, and a strong positive correlation with the same phenomena near the upper plate. In conclusion, the current study reveals that the entropy generation is increased with the Brinkman number and reduced with the squeezing parameter. Moreover, the results of the current study verify and show a decent agreement with the data from earlier published research outcomes.

## 1. Introduction

Squeezing flow is defined as a flow where the material is squeezed out or disfigured in two parallel plates. The flow is useful in various fields, for instance, in welding engineering and rheometry. In addition, utilizing nanofluids in various industrial applications is extensively investigated for augmented heat transfer characteristics. Squeeze flow is an attractive technique for measuring the rheological properties of materials that create difficulties in conventional rheometers, for example, very viscous materials, fluids with an apparent yield stress, fluids that tend to slip at instrument walls, or materials with large particles ( these characteristics often appear together) (Engmann et al. [1]). Su and Yin [2] analyzed the squeezing flow of the fluid among parallel plates with an inclined magnetic field, and witnessed the reduction in the fluid temperature with a larger squeezing number. In addition, Munawar et al. [3] applied a shooting strategy to unriddle the system of equations in their analysis of rotating and squeezed flow amidst parallel plates and discovered that, near the boundaries, a larger magnetic field parameter ameliorates the pressure gradient. Shahmohamadi and Rashidi [4] used the variational iteration (VI) method to provide analytical solutions in the investigation of various nanofluid flows amidst parallel plates, and noticed that the addition of nanoparticles to the fluid exhibits a prominent influence on its velocity. Hayat et al. [5] attempted to investigate the effect of nonlinear radiation on MHD flow over a stretching cylinder. In addition, Khan et al. [6] explicated the characteristics of heat transfer in the water-based nanofluid rotating flow where the solutions are offered by the Runge–Kutta–Fehlberg method. With the assistance of a finite difference scheme, Ahmad et al. [7] numerically scrutinized the nanofluid flow amidst parallel plates with Brownian motion and thermophoresis. They remarked that the fluid velocity is minified with a larger porosity parameter. Al-Kouz et al. [8,9] numerically investigated the rarified nanofluid flow inside a square cavity with fins, and inside a pipe. In addition, Al-Kouz et al. [10] provided an entropy generation optimization study of nanofluids inside a cavity. Moreover, Mahanthesh et al. [11] examined the effect of radiation on nanoliquid flow over a vertical plate. Furthermore, Alshare et al. [12] provided the effect of the wavy configuration module on the flow of nanofluids. Owhaib et al. [13] presented a 3D numerical analysis of rotating nanofluid flow radiation and viscous heating effect using the modified Buongiorno model. Al-Kouz and Owhaib [14] investigated Casson nanofluid heat transfer characteristics over a rotating frame. Atlas et al. [15] studied unsteady Casson nanofluid flow amidst parallel plates with Cattaneo–Christov heat flux. Tarakaramu and Narayana [16] elucidated rotating bioconvective fluid flow among parallel plates with chemical reactions, and observe that the Brownian motion lowers the motile density distribution. Alzahrani et al. [17] examined squeezed flow among parallel plates with cross-diffusion effects, and discovered that the squeezing parameter meliorates fluid temperature. In addition, Khan et al. [18] analyzed the entropy generation optimization in the squeezing flow of second-grade fluid among two parallel plates with thermophoresis and Brownian motion. They emphasized that there is an escalation in the entropy generation with the raise in squeezing parameter. Upreti et al. [19] discussed the optimization of entropy generation in the squeezing flow of hybrid nanofluid with a magnetic field. Zangooee et al. and Salehi et al. [20,21] used the Akbari–Ganji method (AGM) to numerically study various nanofluid rotating flows amidst parallel plates, and acknowledge that the skin friction minifies with a larger Eckert number. Awan et al. [22] employed the Adams P–C method together with R–K4th to solve the mathematical model of the EMHD micropolar fluid flow among parallel plates with Hall current. In addition, Shankar et al. [23] examined the temperature profile in two cases i.e., Fourier’s law model and non-Fourier’s law model (C–C heat flux), and detected that there is a diminution in temperature when the latter is incorporated compared to the former. Magodora et al. [24] applied the spectral quasi-linearization (SQ) method to scrutinize the radiative rotating flow of nanofluid amidst parallel plates with the non-Fourier’s law model, and observed that the nanoparticle volume fraction parameter minimizes the Sherwood number. Recently, Mollah et al. [25] elucidated the rotating flow of Bingham fluid amidst parallel plates with Hall current, and discovered that the velocity profile slowly achieves a steady-state compared with the temperature profile. It is worth mentioning that no study reported in the literature that performs irreversibility analysis in the squeezing flow of hybrid nanofluid between two parallel plates with activation energy and thermal radiation.

Nanofluids were developed as a novel type of heat transfer fluid that may be used in lieu of conventional fluids in industrial operations. They are used in a variety of applications, including refrigeration, heat exchangers, and electronic device cooling. Hybrid nanoparticles are defined as nanoparticles composed of two or more different materials of nanometer size. The fluids prepared with hybrid nanoparticles are known as hybrid nanofluids. Generally, a hybrid nanofluid (HNF) is a superior alternative to a nanofluid (NF). For illustration, silver and copper have more noteworthy thermal conductivities, yet they are flimsy and chemically reactive. By performing the hybridization of such nanoparticles with ceramic or metal oxides, the ensuring HNF shows more prominent rheological behavior and thermo-physical attributes, alongside the developed heat transfer features (Babu et al. [26]). HNF is utilized in various heat transfer applications admitting micropower generation and solar thermal systems. A hybrid nanofluid, which is a combination of a propylene glycol–water mixture and paraffin wax–sand, may be utilized as a standby for the propylene glycol–water blend in the solar thermal framework. Hayat et al. [27] used the shooting method to obtain the results of a study of the ferromagnetic nanomaterial fluid flow of Maxwell fluid on a stretching surface with a magnetic dipole effect. They noticed an improvement in fluid temperature as the ferromagnetic interaction variable increased. The results of Chen et al. [28,29] indicate that the scattering effect, including the scattering ability and scattering phase function, is significant to evaluate the direct solar absorption performance of nanoparticle suspensions, and Cu@C nanoparticle suspensions can be a potential working fluid in solar thermal conversion applications. Furthermore, Qayyum et al. [30] considered different water-based nanofluid flows with Ohmic heating and slip effect, using a rotating disc with variable thickness as a geometry. Their findings include the observation that as the stretching parameter improves, the fluid velocity increases. Moreover, Waini et al. [31] elucidated the nixed convective flow of a water-based hybrid nanofluid using a thin needle and remarked that, near the lower branch, a larger volume fraction nanoparticle of copper ameliorates the temperature. Later, several researchers [32,33,34] considered different stretching surfaces, and analyzed fluid flow with various parameters including radiation and magnetic field. They discovered that the Eckert number and non-linear parameter meliorate fluid temperature. In addition, Khan et al. [35] used Cattaneo–Christov double diffusions to simulate the slip flow of Williamson nanofluid on a permeable stretching surface. One of their findings is that the Williamson parameter can help reduce shear stress. Acharya et al. [36] noticed that the Hall current parameter ameliorates the heat transport in the examination of the radiative flow through a spinning disk. In addition, Eid and Nafe [37] elucidated the flow via an exponentially shrinking/stretching sheet with a heat sink/source. They observed that along with shrinking sheet, copper and magnetite volume fraction nanoparticle parameters exhibit different behaviors on the velocity profile. Also, Khan et al. [38] investigated the non-Darcy flow of micropolar ferrofluid on a permeable stretching sheet in the slip regime using Joule heating and heat generation/absorption. They discovered that the micropolar parameter increases fluid velocity. Abbas et al. [39] numerically simulated hybrid nanofluid (SWCNT–MWCNT/water) flow with bvp4c strategy for two models, Xue and Yamada–Ota, through a thin needle. Ahmad and Nadeem [40] discussed entropy generation optimization in Casson–EG/(SWCNT–MWCNT) fluid flow through a lubricated surface, and detected that the Casson parameter shows mixed behavior on the entropy generation profile. Recently, many authors [41,42,43,44] considered stretching surface and rotating disk, and numerically investigated different hybrid nanofluid flows with various parameters, including Arrhenius energy.

Initially, Menzinger and Wolfgang [44] deliberated the conceptual meaning of Arrhenius activation energy. In addition, Khan et al. [45] used HAM to investigate the effect of chemical reactions on tangent hyperbolic fluid flow on a slender stretching surface with a heat source/sink. They detected a decrease in fluid concentration for larger chemical reaction parameters. Moreover, Devi and Mabood [46] concluded that the activation energy parameter minimizes the Sherwood number in the scrutiny of entropy generation minimization (EGM) on the Maxwell fluid flow through a rotating disk with the Marangoni model. Furthermore, Kumar et al. [47] explained the features of heat transfer in the tangent hyperbolic fluid flow on an elongated sheet with activation energy and thermophoresis. In addition, Bhatti and Michaelides [48] used Mathematica software to offer numerical solutions to the bioconvective nanofluid flow on a Riga plate, and observed a diminution in fluid concentration with a larger reaction rate parameter. Khan and Alzahrani [49] inspected the entropy generation optimization in the dissipative flow of Jeffrey nanofluid on a curved stretched surface with thermophoresis and activation energy. In addition, Irfan et al. [50] explained the characteristics of the mass flux concept with activation energy on the blended convective flow of Carreau fluid. Also, Wang et al. [51] investigated the effect of homogeneous and heterogeneous reactions on the dissipative flow of an Oldroyd-B fluid over a convectively heated surface with a heat source/sink. They discovered that increasing the heterogeneous parameter improves fluid concentration. Recently, many researchers [52,53,54,55] considered different geometries and demonstrated various fluid flows with activation energy, and detected that the Reynolds number minimizes the tangential velocity.

The modeling of rotating flow is critically important across a wide range of scientific, engineering, and product design applications, providing design capability for products such as jet engines, pumps, and vacuum cleaners, and modeling capability for geophysical flows. Even for applications where rotation is not initially evident, the subject is often fundamental to understanding and modeling the details of the flow physics. Examples include the vorticity produced in flow along a channel, the secondary flow produced for flow around a bend, and the wing-tip vortices produced downstream of a wing [56]. Hayat et al. [57] found that the temperature profile increases by increasing the rotation parameter in their analysis on the rotating flow of an $Ag-CuO/H_{2}O$ hybrid nanofluid with radiation and partial slip boundary effects. Shoaib et al. [58] numerically investigated the rotating flow of hybrid nanofluid over a stretchable sheet with thermal radiation, and noticed a reduction in the velocity field with the rise in rotation parameter. Recently, Lie et al. [59] and Mohd Sohut et al. [60] discussed the rotating flows of various hybrid nanofluids over a stretching sheet under different conditions. In addition to the previously mentioned references, more work related to nanofluids and nanogeometries can be found in [61,62,63].

It is noticed that no study found in the literature was conducted on the unsteady radiative squeezing flow of hybrid nanofluid between two parallel plates with irreversibility analysis. Hence, the current paper’s objective is to investigate such flow with a propylene glycol–water-based hybrid nanofluid with viscous dissipation. The Arrhenius energy equation is integrated to explain mass transport phenomena. Impressions of diverse parameters on the flow are demonstrated by expending tables and graphs. Results are verified with the earlier outcomes, and an acceptable accord is noticed. The main goal of this research is to provide answers to the following related research questions:

1. How important is the activation energy in binary nanofluid flow versus mono nanofluid flow?
2. What effect does the thermal radiation parameter have on the binary hybrid nanofluid flow when positive and negative squeezing numbers are taken into account?
3. What effect does the Brinkmann number have on entropy generation in two cases, binary and mono nanofluid flows?
4. Is the reduction of shear stress near the surface is an important task in fluid flow problems?
5. Is the magnetic field parameter relevant to this phenomenon?

## 2. Formulation

**Figure 1:**
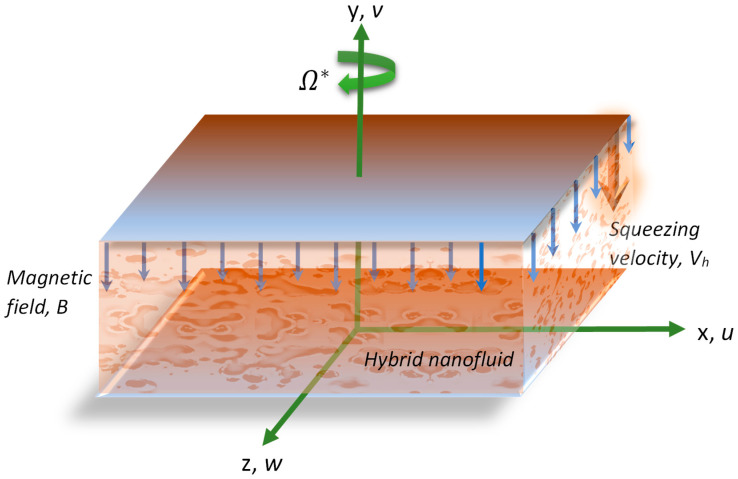
Flow illustration of hybrid nanofluid among two rotating parallel plates.

We considered an incompressible, unsteady, three-dimensional squeezing flow of a hybrid nanofluid amidst (two) parallel plates. We took propylene glycol–water mixture as a base fluid and paraffin wax and sand as nanomaterials, and exhibited the values of their thermophysical attributes in Table 1. Presumptions for the formulation are:

- Higher plate is positioned at y=h(t) and lower plate is placed at y=0, where $h\left(t\right)=\sqrt{\frac{\left(1-\gamma t\right)\upsilon_{f}}{a}}$;
- Lower plate is elongated with the velocity $u_{w}=\frac{ax}{1-\gamma t}.$;
- Magnetic field of intensiveness $B\left(t\right)=\frac{B_{0}}{\sqrt{1-\gamma t}}$ is applied normal to the flow (see Figure 1);
- Squeezing velocity and angular velocity of the fluid are $V_{h}=\frac{dh}{dt}$ and $\Omega *=\frac{\omega }{1-\gamma t}$;
- $T_{w}$ and $T_{h}$ are the temperatures of lower and upper plates, respectively;
- Nanoparticles and base fluid are supposed to be in equilibrium. and no-slip arises amongst them;
- Neglected induced magnetic field and Joule heating.

With these premises, the conservation of mass Equation (1), conservation of momentum Equations (2)–(4), conservation of energy Equation (5), and diffusion Equation (6) are given as (Munawar et al. [3], Alzahrani et al. [17], Anantha Kumar et al. [64], and Irfan et al. [50], respectively):

(1) $$\frac{\partial u}{\partial x}+\frac{\partial v}{\partial y}=0$$

(2) $$\frac{\partial u}{\partial t}+u\frac{\partial u}{\partial x}+v\frac{\partial u}{\partial y}+\frac{2\omega }{1-\gamma t}w=-\frac{1}{\rho_{hnf}}\frac{\partial p}{\partial x}+\upsilon_{hnf}\left(\frac{\partial^{2}u}{\partial x^{2}}+\frac{\partial^{2}u}{\partial y^{2}}\right)-\frac{\sigma B^{2}\left(t\right)}{\rho_{hnf}}u$$

(3) $$\frac{\partial v}{\partial t}+u\frac{\partial v}{\partial x}+v\frac{\partial v}{\partial y}=-\frac{1}{\rho_{hnf}}\frac{\partial p}{\partial y}+\upsilon_{hnf}\left(\frac{\partial^{2}v}{\partial x^{2}}+\frac{\partial^{2}v}{\partial y^{2}}\right)$$

(4) $$\frac{\partial w}{\partial t}+u\frac{\partial w}{\partial x}+v\frac{\partial w}{\partial y}-\frac{2\omega }{1-\gamma t}u=\upsilon_{hnf}\left(\frac{\partial^{2}w}{\partial x^{2}}+\frac{\partial^{2}w}{\partial y^{2}}\right)-\frac{\sigma B^{2}\left(t\right)}{\rho_{hnf}}w$$

(5) $$\begin{array}{l} \frac{\partial T}{\partial t}+u\frac{\partial T}{\partial x}+v\frac{\partial T}{\partial y}=\frac{k_{hnf}}{{\left(\rho C_{p}\right)}_{hnf}}\left(\frac{\partial^{2}T}{\partial x^{2}}+\frac{\partial^{2}T}{\partial y^{2}}\right)-\frac{1}{{\left(\rho C_{p}\right)}_{hnf}}\frac{\partial q_{r}}{\partial y}\\ +\frac{Q_{0}}{{\left(\rho C_{p}\right)}_{hnf}}\left(T-T_{h}\right) \end{array}$$

(6) $$\frac{\partial C}{\partial t}+u\frac{\partial C}{\partial x}+v\frac{\partial C}{\partial y}=D_{m}\left(\frac{\partial^{2}C}{\partial x^{2}}+\frac{\partial^{2}C}{\partial y^{2}}\right)-k_{r}{\left(\frac{T}{T_{h}}\right)}^{n}Exp\left(-\frac{E_{a}}{k_{1}T}\right)\left(C-C_{h}\right)$$

With the boundary conditions (Khan et al. [6]):

(7) $$\begin{array}{l} u=u_{w},v=-\frac{-V_{0}}{1-\gamma t},w=0,T=T_{w},C=C_{w}\;at\;y=0\\ u=0,v=V_{h}=\frac{dh}{dt},w=0,T=T_{h},C=C_{h}\;at\;y=h\left(t\right) \end{array}\}$$

In Equation (5), $q_{r}$ is taken to examine the heat transport performance and it can be defined as:

(8) $$q_{r}=-\frac{4\sigma *}{3k*}\left(\frac{\partial^{2}}{\partial x^{2}}+\frac{\partial^{2}}{\partial y^{2}}\right)T^{4}$$

The Taylor series expansion of $T^{4}$ in terms of $T_{h}$ is (after ignoring higher order terms):

(9) $$T^{4}\approx 4T_{h}^{3}T-3T_{h}^{4}$$

Using (8) and (9), Equation (5) can be rewritten as:

(10) $$\begin{array}{l} \frac{\partial T}{\partial t}+u\frac{\partial T}{\partial x}+v\frac{\partial T}{\partial y}=\frac{k_{hnf}}{{\left(\rho C_{p}\right)}_{hnf}}\left(\frac{\partial^{2}T}{\partial x^{2}}+\frac{\partial^{2}T}{\partial y^{2}}\right)+\frac{1}{{\left(\rho C_{p}\right)}_{hnf}}\frac{16\sigma *T_{h}^{3}}{3k*}\left(\frac{\partial^{2}T}{\partial x^{2}}+\frac{\partial^{2}T}{\partial y^{2}}\right)+\\ \frac{Q_{0}}{{\left(\rho C_{p}\right)}_{hnf}}\left(T-T_{h}\right) \end{array}$$

### 2.1. Thermophysical Attributes of Hybrid Nanofluid

Here, $\phi_{1}$ and $\phi_{2}$ are the nanoparticle volume fractions, subscripts hnf and nf indicate hybrid and mono nanofluids, respectively, f indicates base fluid, and $s_{1}$ and $s_{2}$ specify nanomaterials.


$$\begin{array}{l} \rho_{hnf}=\left(1-\phi_{2}\right)\left[\left(1-\phi_{1}\right)\rho_{f}+\phi_{1}\rho_{s_{1}}\right]+\phi_{2}\rho_{s_{2}},\mu_{hnf}=\frac{\mu_{f}}{{\left(1-\phi_{1}\right)}^{2.5}{\left(1-\phi_{2}\right)}^{2.5}},\\ \sigma_{hnf}=\frac{\sigma_{s_{2}}+2\sigma_{nf}-2\phi_{2}\left(\sigma_{nf}-\sigma_{s_{2}}\right)}{\sigma_{s_{2}}+2\sigma_{nf}+\phi_{2}\left(\sigma_{nf}-\sigma_{s_{2}}\right)}\times \sigma_{nf},\sigma_{nf}=\frac{\sigma_{s_{1}}+2\sigma_{f}-2\phi_{1}\left(\sigma_{f}-\sigma_{s_{1}}\right)}{\sigma_{s_{1}}+2\sigma_{f}+\phi_{1}\left(\sigma_{f}-\sigma_{s_{1}}\right)}\times \sigma_{f}\\ k_{hnf}=\frac{k_{s_{2}}+2k_{nf}-2\phi_{2}\left(k_{nf}-k_{s_{2}}\right)}{k_{s_{2}}+2k_{nf}+\phi_{2}\left(k_{nf}-k_{s_{2}}\right)}\times k_{nf},k_{nf}=\frac{k_{s_{1}}+2k_{f}-2\phi_{1}\left(k_{f}-k_{s_{1}}\right)}{k_{s_{1}}+2k_{f}+\phi_{1}\left(k_{f}-k_{s_{1}}\right)}\times k_{f}\\ {\left(\rho C_{p}\right)}_{hnf}=\left(1-\phi_{2}\right)\left[\left(1-\phi_{1}\right){\left(\rho C_{p}\right)}_{f}+\phi_{1}{\left(\rho C_{p}\right)}_{s_{1}}\right]+\phi_{2}{\left(\rho C_{p}\right)}_{s_{2}} \end{array}\}$$

**Table 1:** Thermophysical properties of nanomaterials and base fluid (Reprinted with permission from Ref. [65]. Copyright 2022 Copyright Elsevier).

S. No.		Propylene Glycol—Water Mixture (f)	Paraffin Wax $\left(s_{1}\right)$	Sand $\left(s_{2}\right)$
**1**	$\rho \left(Kg/m^{3}\right)$	1020	900	2650
**2**	$C_{p}\left(J/Kg\;K\right)$	3342.75	2900	730
**3**	k(W/m K)	0.363	0.25	1.5

### 2.2. Similarity Transmutations 

Similarity transmutations (Khan et al. [66]) in Equation (11) as:

(11) $$\begin{array}{l} \eta =\frac{y}{h\left(t\right)},u=\frac{ax}{1-\gamma t}f^{\prime },v=-\sqrt{\frac{a\upsilon }{1-\gamma t}}f,\\ w=\frac{ax}{1-\gamma t}g,\theta =\frac{T-T_{h}}{T_{w}-T_{h}},\Phi =\frac{C-C_{h}}{C_{w}-C_{h}} \end{array}\}$$

### 2.3. Transmuted Equations and Conditions

Transmutations satisfies Equation (1), and alters Equations (2)–(4), (6) and (10) as:

(12) $$\frac{1}{H_{1}H_{2}}f^{\prime \prime \prime }-\frac{1}{2}S_{q}\eta f^{\prime \prime }-S_{q}f^{\prime }-{f^{\prime }}^{2}+ff^{\prime \prime }-2\Omega g+\frac{M}{H_{1}}f^{\prime }=-\frac{{\left(1-\gamma t\right)}^{2}}{a^{2}x}\frac{1}{\rho_{f}H_{1}}\frac{\partial p}{\partial x}$$

(13) $$f^{\prime \prime }-\frac{1}{2}S_{q}\eta f^{\prime }-\frac{S_{q}}{2}f+ff^{\prime }=-\frac{a}{{\left(1-\gamma t\right)}^{2}}\frac{1}{\rho_{f}H_{1}}\frac{\partial p}{\partial \eta }$$

(14) $$\frac{1}{H_{1}H_{2}}g^{\prime \prime }-\frac{1}{2}S_{q}\eta g^{\prime }-S_{q}g+fg^{\prime }-f^{\prime }g+2\Omega f^{\prime }-\frac{M}{H_{1}}g=0$$

(15) $$\left(\frac{R_{a}+H_{3}H_{31}}{H_{4}}\right)\frac{\theta^{\prime \prime }}{\mathrm{Pr}}+f\theta^{\prime }-\frac{\eta }{2}S_{q}\theta^{\prime }+\frac{N}{H_{4}}\theta =0$$

(16) $$\frac{1}{Sc}\Phi^{\prime \prime }+f\Phi^{\prime }-\frac{S_{q}}{2}\eta \Phi^{\prime }-\Gamma {\left(1+\alpha_{1}\theta \right)}^{n}Exp\left(-\frac{E_{n}}{1+\alpha_{1}\theta }\right)\Phi =0$$

and alters the conditions in Equation (7) as:

(17) $$\begin{array}{l} f(0)=S,f^{\prime }(0)=1,g\left(0\right)=1,\theta \left(0\right)=1,\Phi \left(0\right)=1\;\\ f\left(1\right)=\frac{S_{q}}{2},f^{\prime }\left(1\right)=0,g\left(1\right)=0,\;\theta \left(1\right)=0,\Phi \left(1\right)=0 \end{array}\}$$

where

$$\begin{array}{l} H_{1}={\left(1-\phi_{1}\right)}^{2.5}{\left(1-\phi_{2}\right)}^{2.5},H_{2}=\left(1-\phi_{2}\right)\left[\left(1-\phi_{1}\right)+\phi_{1}\frac{\rho_{s_{1}}}{\rho_{f}}\right]+\phi_{2}\frac{\rho_{s_{2}}}{\rho_{f}},H_{31}=\frac{k_{s_{1}}+2k_{f}-2\phi_{1}\left(k_{f}-k_{s_{1}}\right)}{k_{s_{1}}+2k_{f}+\phi_{1}\left(k_{f}-k_{s_{1}}\right)},\\ H_{3}=\frac{k_{s_{2}}+2H_{31}k_{f}-2\phi_{2}\left(H_{31}k_{f}-k_{s_{2}}\right)}{k_{s_{2}}+2H_{31}k_{f}+\phi_{2}\left(H_{31}k_{f}-k_{s_{2}}\right)},H_{4}=\left(1-\phi_{2}\right)\left[\left(1-\phi_{1}\right)+\phi_{1}\frac{{\left(\rho C_{p}\right)}_{s_{1}}}{{\left(\rho C_{p}\right)}_{f}}\right]+\phi_{2}\frac{{\left(\rho C_{p}\right)}_{s_{2}}}{{\left(\rho C_{p}\right)}_{f}} \end{array}\}.$$

and

$$\begin{array}{l} S_{q}=\frac{d}{a},\Omega =\frac{\omega }{a},M=\frac{\sigma B_{0}^{2}}{\rho_{f}a},\mathrm{Pr}=\frac{\mu {\left(C_{p}\right)}_{f}}{k_{f}},R_{a}=\frac{16}{3}\frac{\sigma *T_{h}^{3}}{k_{f}k*},\\ N=\frac{Q_{0}\left(1-dt\right)}{a{\left(\rho C_{p}\right)}_{f}},\Gamma =\frac{k_{r}\left(1-dt\right)}{a},\alpha_{1}=\frac{T_{w}-T_{h}}{T_{h}},E_{n}=\frac{E_{a}}{k_{1}T_{h}},S=\frac{V_{0}}{ah} \end{array}\}$$

Applied cross-differentiation on Equations (12) and (13) leads to a simplified fourth-order differential, where the similarity solution is maintained, the number of the independent variables is reduced, and the pressure term from Equations (2) and (3) is disregarded. The consequential equation is:

(18) $$\frac{1}{H_{1}H_{2}}f^{iv}-\frac{S_{q}}{2}\left(\eta f^{\prime \prime \prime }+3f^{\prime \prime }\right)-f^{\prime }f^{\prime \prime }+ff^{\prime \prime \prime }-2\Omega g^{\prime }-\frac{M}{H_{1}}f^{\prime \prime }=0$$

### 2.4. Physical Parameters

Near the lower plate, surface drag force is defined as (Alzahrani et al. [17]):

(19) $$C_{f_{0}}=\frac{{\mu_{hnf}\left(\frac{\partial v}{\partial x}+\frac{\partial u}{\partial y}\right)|}_{y=0}}{\rho_{hnf}u_{w}^{2}},C_{g_{0}}=\frac{{\mu_{hnf}\left(\frac{\partial w}{\partial y}\right)|}_{y=0}}{\rho_{hnf}u_{w}^{2}}$$

Near the upper plate, the same (surface drag force) is defined as:

(20) $$C_{f_{1}}=\frac{{\mu_{hnf}\left(\frac{\partial v}{\partial x}+\frac{\partial u}{\partial y}\right)|}_{y=h\left(t\right)}}{\rho_{hnf}u_{w}^{2}},C_{g_{1}}=\frac{{\mu_{hnf}\left(\frac{\partial w}{\partial y}\right)|}_{y=h\left(t\right)}}{\rho_{hnf}u_{w}^{2}}$$

With the aid of Equations (11), (19) and (20), it can be rewritten in dimensionless form as:

(21) Cf0=1H1H21Rexf″(0),Cg0=1H1H21Rexg′(0)

and

(22) Cf1=1H1H21Rexf″(1),Cg1=1H1H21Rexg′(1)

where Rex=xuwυf (local Reynolds number).

Formulae to find transfer rates (heat and mass) (or Nusselt and Sherwood numbers) near lower and upper plates are:

(23) $$Nu_{0}=\frac{x{q_{w}|}_{y=0}}{k_{f}\left(T_{w}-T_{h}\right)},Nu_{1}=\frac{x{q_{w}|}_{y=h\left(t\right)}}{k_{f}\left(T_{w}-T_{h}\right)}\;\mathrm{and}Sh_{0}=\frac{x{s_{w}|}_{y=0}}{D_{m}\left(C_{w}-C_{h}\right)},Sh_{1}=\frac{{xs_{w}|}_{y=h\left(t\right)}}{D_{m}\left(C_{w}-C_{h}\right)}$$

where the wall heat flux, $q_{w}=-(K_{\mathrm{hnf}}+\frac{16\sigma *T_{h}^{3}}{3k*})\frac{\partial T}{\partial y}$ and the wall mass flux,

(24) $$s_{w}=-D_{m}\frac{\partial C}{\partial y}$$

With the aid of Equations (8) and (24), formulae in (23) can be rewritten as:

$$Nu_{0}=-\left(\frac{k_{hnf}}{k_{f}}+R_{a}\right)\theta^{\prime }\left(0\right),Nu_{1}=-\left(\frac{k_{hnf}}{k_{f}}+R_{a}\right)\theta^{\prime }\left(1\right)$$

and

$$Sh_{0}=-\Phi^{\prime }\left(0\right),Sh_{1}=-\Phi^{\prime }\left(1\right)$$

### 2.5. Entropy Generation and Bejan Number

The volumetric rate of entropy generation (dimensional form) for the hybrid nanofluid flow among two parallel plates is specified as:

(25) $$\begin{array}{l} S_{gen}=\frac{k_{hnf}}{T_{h}^{2}}\left[{\left(\frac{\partial T}{\partial x}\right)}^{2}+{\left(\frac{\partial T}{\partial y}\right)}^{2}\right]+\frac{1}{T_{h}^{2}}\frac{16}{3}\frac{\sigma *T_{\infty }^{3}}{k*}\left[{\left(\frac{\partial T}{\partial x}\right)}^{2}+{\left(\frac{\partial T}{\partial y}\right)}^{2}\right]+\frac{\mu_{hnf}}{T_{h}}\varphi +\frac{\sigma {\left(B\left(t\right)\right)}^{2}}{T_{2}}\left(u^{2}+w^{2}\right)\\ +\frac{\bar{R}D_{m}}{C_{h}}\left[{\left(\frac{\partial C}{\partial x}\right)}^{2}+{\left(\frac{\partial C}{\partial y}\right)}^{2}\right]+\frac{\bar{R}D_{m}}{T_{h}}\left(\frac{\partial C}{\partial x}\frac{\partial T}{\partial x}+\frac{\partial C}{\partial y}\frac{\partial T}{\partial y}\right) \end{array}$$

where

(26) $$\varphi =4{\left(\frac{\partial u}{\partial x}\right)}^{2}+{\left(\frac{\partial v}{\partial x}+\frac{\partial u}{\partial y}\right)}^{2}+{\left(\frac{\partial w}{\partial x}\right)}^{2}+{\left(\frac{\partial w}{\partial y}\right)}^{2}$$

and $\bar{R}$ is the universal gas constant.

The non-dimensional form of Equation (25) is:

(27) $$\begin{array}{l} N_{EG}=\left(H_{3}H_{31}+R_{a}\right)\alpha_{1}{\theta^{\prime }}^{2}+Br\left(4{f^{\prime }}^{2}+g^{2}\right)+Br_{1}\left({f^{\prime \prime }}^{2}+{g^{\prime }}^{2}\right)+\frac{BrM}{H_{2}}\left({f^{\prime }}^{2}+g^{2}\right)\\ +H*\frac{\alpha_{2}}{\alpha_{1}}{\Phi^{\prime }}^{2}+H*\Phi^{\prime }\theta^{\prime } \end{array}$$

Entropy generation $N_{EG}$, Brinkman number Br, local Brinkman number $Br_{1}$, diffusion parameter H*, and the concentration ratio parameter $\alpha_{2}$ are specified as:

$$\begin{array}{l} N_{EG}=\frac{\upsilon \left(1-dt\right)T_{h}S_{G}}{a\left(T_{w}-T_{h}\right)k_{f}},Br=\frac{\mu \upsilon^{2}}{h^{2}\left(T_{w}-T_{h}\right)k_{f}},Br_{1}=\frac{\mu u_{w}^{2}}{\left(T_{w}-T_{h}\right)k_{f}},\\ H*=\frac{\bar{R}D_{m}\left(C_{w}-C_{h}\right)}{k_{f}},\alpha_{2}=\frac{C_{w}-C_{h}}{C_{h}} \end{array}\}$$

By using the below formula, we can evaluate the Bejan number:

$$Be=\frac{\mathrm{Entropy}\;\mathrm{generation}\;\mathrm{on}\;\mathrm{account}\;\mathrm{of}\;\mathrm{heat}\;\mathrm{and}\;\mathrm{mass}\;\mathrm{transfer}}{\mathrm{Total}\;\mathrm{entropy}\;\mathrm{generation}}$$

By using Equation (27), Be can be articulated as

$$Be=\frac{\left(H_{3}H_{31}+R_{a}\right)\alpha_{1}{\theta^{\prime }}^{2}+H*\frac{\alpha_{2}}{\alpha_{1}}{\Phi^{\prime }}^{2}+H*\Phi^{\prime }\theta^{\prime }}{\left(H_{3}H_{31}+R_{a}\right)\alpha_{1}{\theta^{\prime }}^{2}+Br\left(4{f^{\prime }}^{2}+g^{2}\right)+Br_{1}\left({f^{\prime \prime }}^{2}+{g^{\prime }}^{2}\right)+\frac{BrM}{H_{2}}({f^{\prime }}^{2}+g^{2})+H*\frac{\alpha_{2}}{\alpha_{1}}{\Phi^{\prime }}^{2}+H*\Phi^{\prime }\theta^{\prime }}.$$

## 3. Numerical Procedure

The fourth-order Runge–Kutta method and shooting procedure combination are engaged to solve Equations (14)–(16) and (18), with the conditions presented in Equation (17).

Let

$$x_{1}=f,x_{2}=f^{\prime },x_{3}=f^{\prime \prime },x_{4}=f^{\prime \prime \prime },x_{5}=g,x_{6}=g^{\prime },x_{7}=\theta ,x_{8}=\theta^{\prime },x_{9}=\Phi ,x_{10}=\Phi^{\prime }$$

Then, using Equations (11)–(13) and (15), we can develop the subsequent system of ODEs of the first-order:

(28) $$\begin{array}{l} {x_{1}}^{\prime }=x_{2},\\ {x_{2}}^{\prime }=x_{3},\\ {x_{3}}^{\prime }=x_{4},\\ {x_{4}}^{\prime }=H_{1}H_{2}\left[\frac{S_{q}}{2}\left(\eta x_{4}+3x_{3}\right)+x_{2}x_{3}-x_{1}x_{4}+2\Omega x_{6}+\frac{M}{H_{1}}x_{3}\right],\\ {x_{5}}^{\prime }=x_{6},\\ {x_{6}}^{\prime }=H_{1}H_{2}\left[\frac{S_{q}}{2}\eta x_{6}+S_{q}x_{5}+x_{2}x_{5}-x_{1}x_{6}-2\Omega x_{2}+\frac{M}{H_{1}}x_{5}\right],\\ {x_{7}}^{\prime }=x_{8}\\ {x_{8}}^{\prime }=-\mathrm{Pr}\left(\frac{H_{4}}{R_{a}+H_{3}H_{31}}\right)\left[x_{1}x_{8}-\frac{\eta }{2}S_{q}x_{8}+\frac{1}{H_{2}H_{4}}\left[E_{c}\left(4x_{2}^{2}+x_{6}^{2}\right)+E_{cx}\left(x_{3}^{2}+x_{6}^{2}\right)\right]+Nx_{7}\right]\\ {x_{9}}^{\prime }=x_{10}\\ {x_{10}}^{\prime }=-Sc\left[x_{1}x_{10}-\frac{S_{q}}{2}\eta x_{10}-\Gamma {\left(1+\alpha_{1}x_{7}\right)}^{n}Exp\left(-\frac{E_{n}}{1+\alpha_{1}x_{7}}\right)x_{9}\right] \end{array}\}$$

with the initial conditions:

(29) $$\begin{array}{l} x_{1}\left(0\right)=S,x_{2}\left(0\right)=1,x_{3}\left(0\right)=\varsigma_{1},x_{4}\left(0\right)=\varsigma_{2},x_{5}\left(0\right)=1\\ x_{6}\left(0\right)=\varsigma_{3},x_{7}\left(0\right)=1,x_{8}\left(0\right)=\varsigma_{4},x_{9}\left(0\right)=1,x_{10}\left(0\right)=\varsigma_{5} \end{array}\}$$

Here $\varsigma_{1},\varsigma_{2},\varsigma_{3},\varsigma_{4}$, and $\varsigma_{5}$ are the required introductory guesses to sort out the solution. The fourth-order R–K scheme is imposed to obtain the solution. Afterward, we calculate $x_{1}\left(1\right),x_{2}\left(1\right),x_{5}\left(1\right),x_{7}\left(1\right),x_{9}\left(1\right)$ values, and compare them with the current values of the equivalent. If they are not appropriately equivalent, using the shooting strategy to change the estimations $x_{3}\left(0\right),x_{4}\left(0\right),x_{6}\left(0\right),x_{8}\left(0\right),x_{10}\left(0\right)$ obtains a decent solution. This procedure will be repeated until we achieve the desired precision.

## 4. Validation

We verified our outcomes with the earlier results for friction factor (with two methods) under special circumstances, such as $\phi_{1}=\phi_{2}=0$ and M=0.5, and detected an acceptable agreement (see Table 2).

## 5. Discussion

The impacts of pertinent parameters on the flow are explained in two situations. The first situation dealt with the comparison of two cases, i.e., hybrid and mono nanofluids, and the second situation dealt with the comparison of two cases, i.e., positive squeezing parameter (upper plate proceeds in the direction of the lower plate) and negative squeezing parameter (upper plate is moving apart from the lower plate).

### 5.1. Velocity Profiles

**Figure 2:**
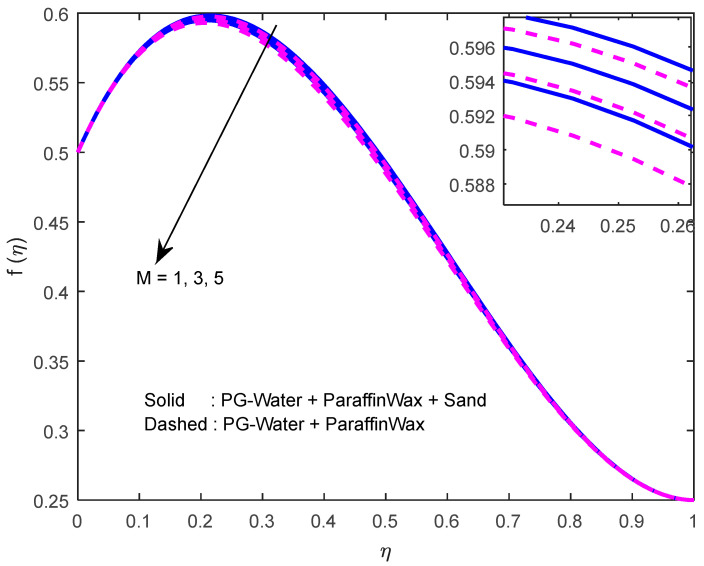
Effect of the magnetic field parameter on the velocity profile in y-direction.

**Figure 3:**
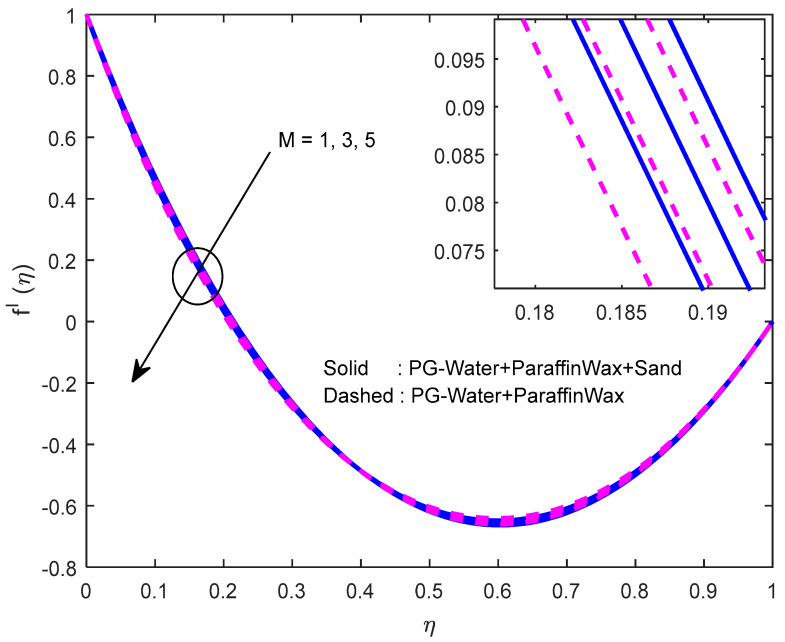
Effect of the magnetic field parameter on the velocity profile in x-direction.

**Figure 4:**
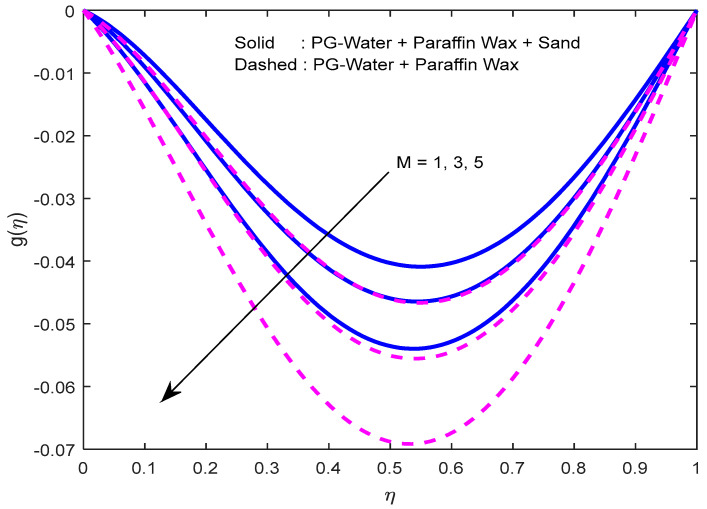
Effect of the magnetic field parameter on velocity profile in z-direction.

**Figure 5:**
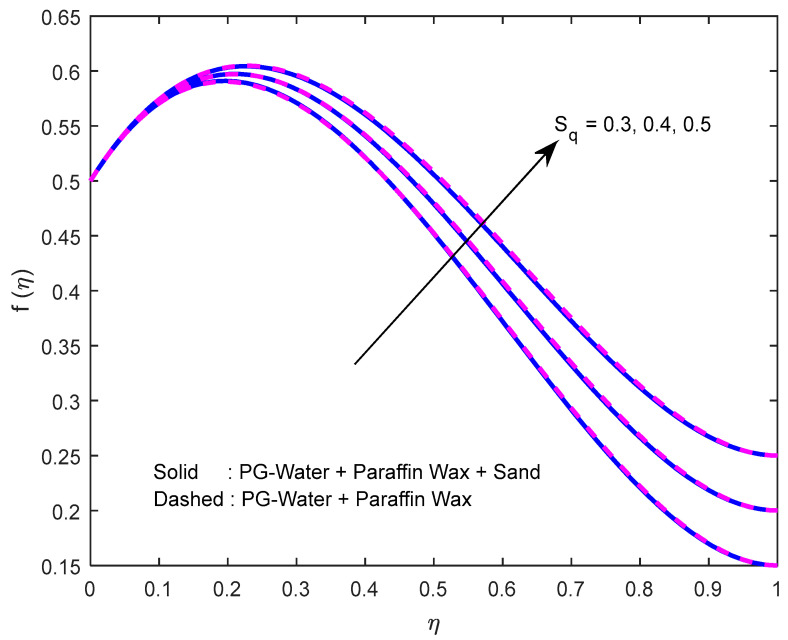
Effect of the squeeze number on velocity profile in y-direction.

**Figure 6:**
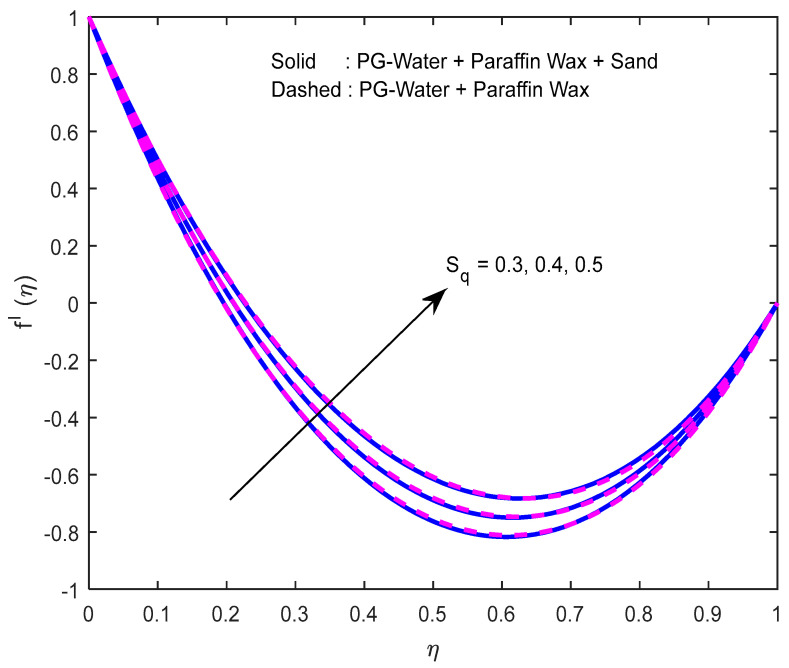
Effect of the squeeze number on the velocity profile in x -direction.

**Figure 7:**
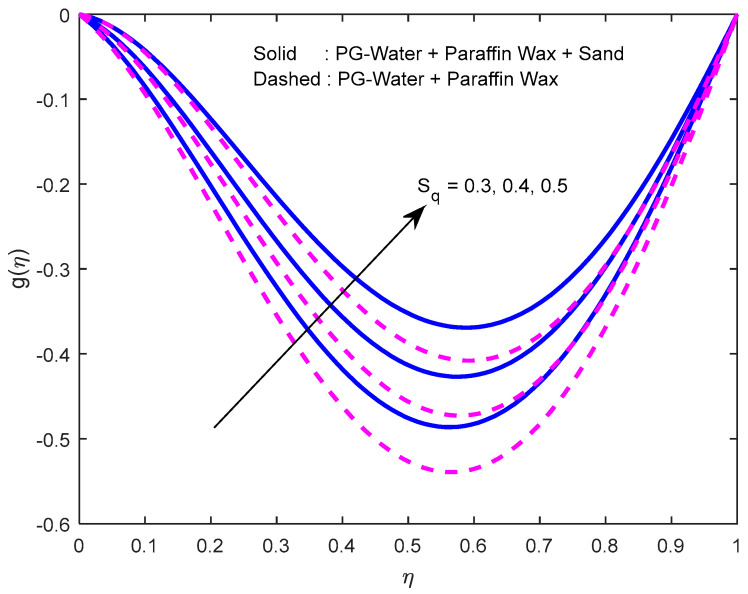
Effect of the squeeze number on the velocity profile in z-direction.

**Figure 8:**
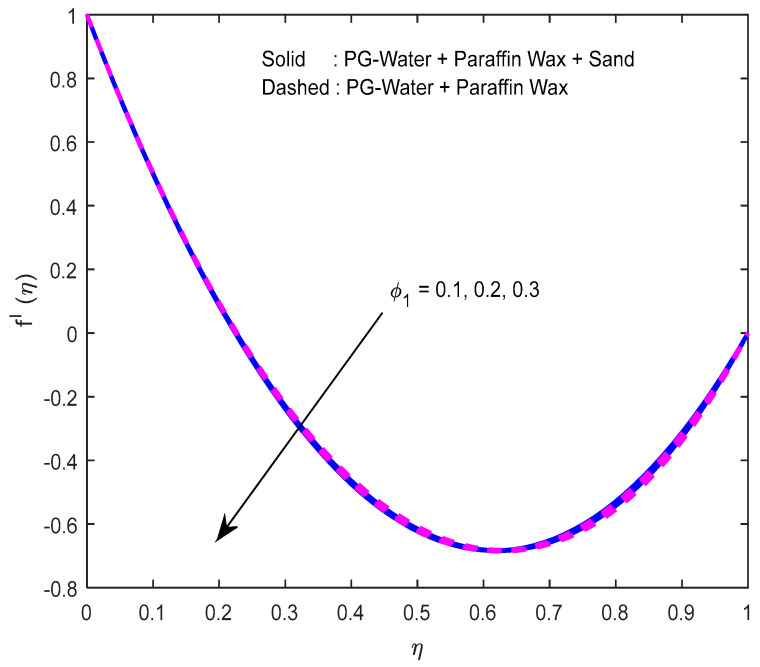
Effect of the nanoparticle volume fraction parameter on the velocity profile in x-direction.

**Figure 9:**
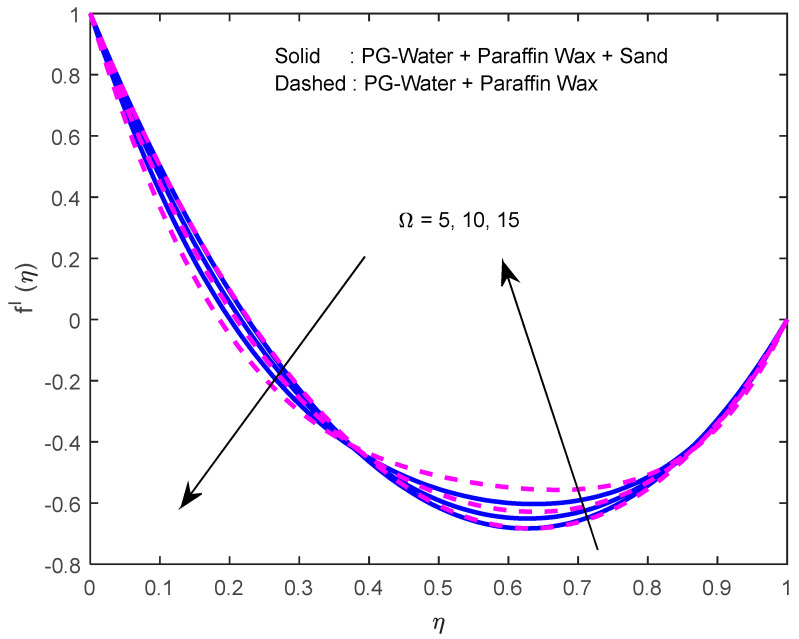
Effect of the rotation parameter on the velocity profile in x-direction.

Fluid particles try to change their direction within the sight of the magnetic field. So, the velocity of the fluid deprecates with a larger magnetic field (Figure 2, Figure 3 and Figure 4). Figure 5, Figure 6 and Figure 7 account for the impact of the squeezing number $S_{q}$ on the velocity profiles. Generally, with the elevation in squeezing number, there is an increment in the pressure of the fluid. As a result, intensification in fluid velocity occurs. It is evident from Figure 8 that the volumetric nanoparticle parameter alleviates the normal velocity in x-direction because of the enhancement in fluid viscosity. Since the particles move from the lower plate to the upper plate, initially, the concentration of particles is higher at the lower plate than at the upper plate. So, the larger rotation parameter deprecates normal velocity (in x-direction) in the lower half, and ameliorates the same in the upper half (Figure 9).

### 5.2. Temperature Profiles

**Figure 10:**
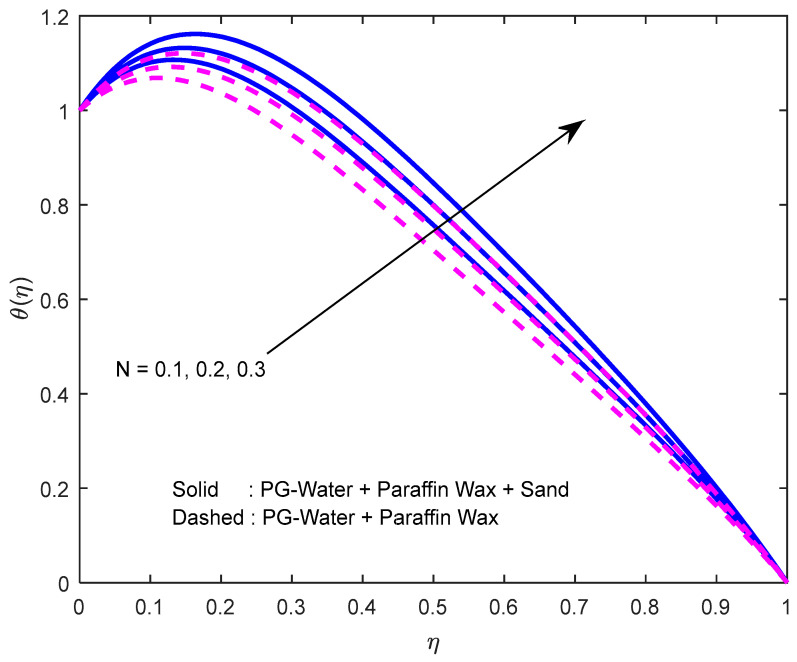
Effect of the heat source parameter on the temperature profile.

**Figure 11:**
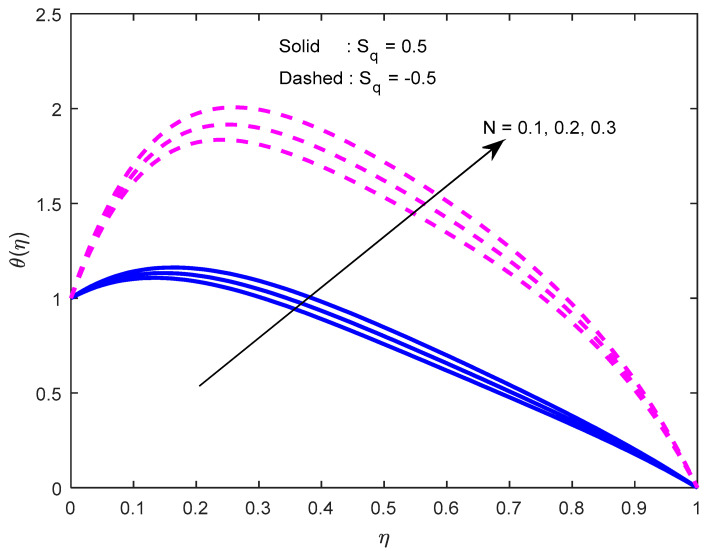
Effect of the heat source parameter on the temperature profile, with varying squeeze numbers.

**Figure 12:**
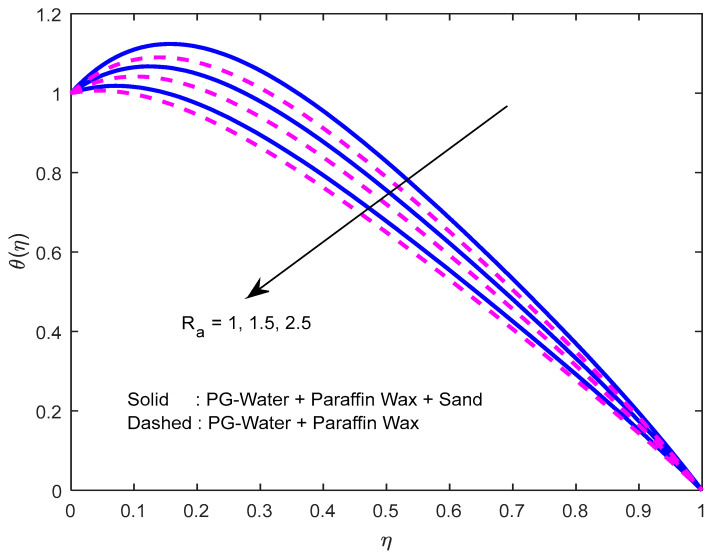
Effect of radiation parameter on the temperature profile.

**Figure 13:**
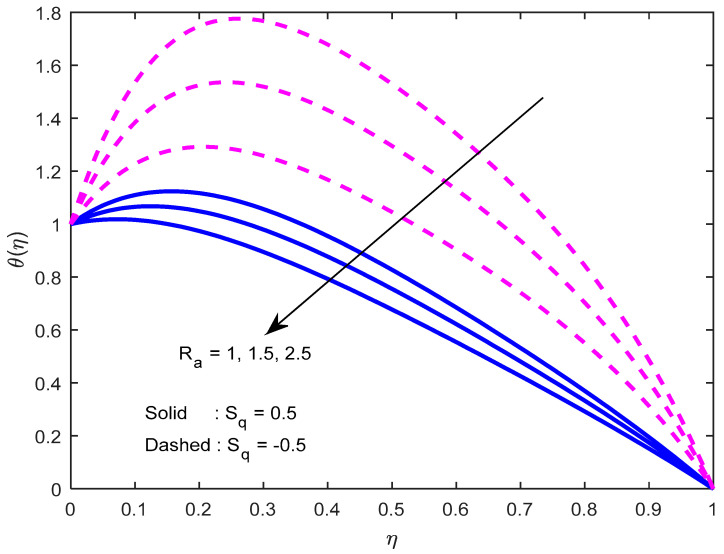
Effect of the radiation parameter on the temperature profile, with varying squeezing numbers.

**Figure 14:**
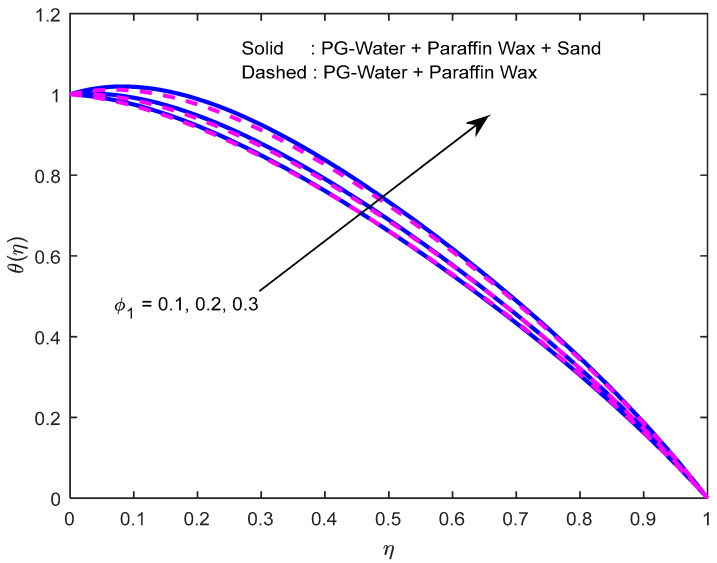
Effect of the nanoparticle volume fraction parameter on the temperature profile.

Figure 10 and Figure 11 exhibit the impression of the heat source parameter N on the temperature profile. It is seen that it escalates temperature. Typically, a larger heat source parameter causes the proliferation of additional heat within the fluid and, in turn, enhances the thickness of the thermal boundary layer. The radiation parameter mitigates fluid temperature (Figure 12 and Figure 13). From these outcomes, it is interesting to remark that the mono nanofluid profile is high in contrast with the hybrid nanofluid profile in the second situation, and the complete opposite behavior observed in the first situation. Larger volumetric nanoparticle parameters generate more friction among particles, which leads to the escalation in fluid temperature (Figure 14).

### 5.3. Concentration Profiles

**Figure 15:**
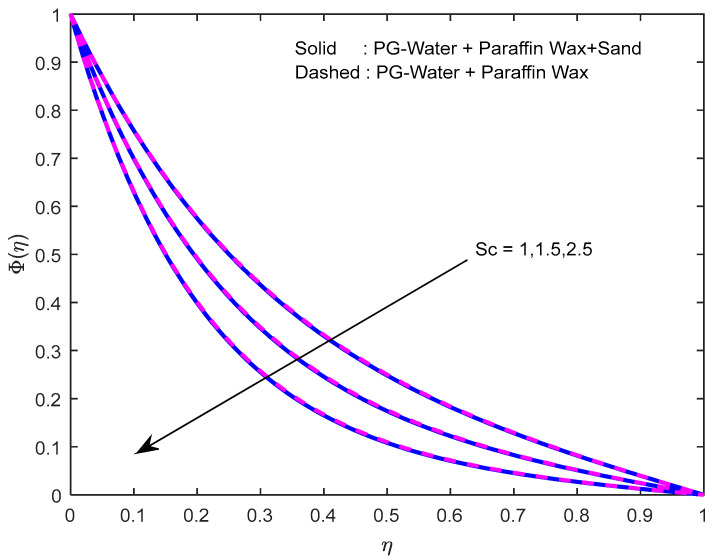
Effect of the Schmidt number on the concentration profile for various nanofluids.

**Figure 16:**
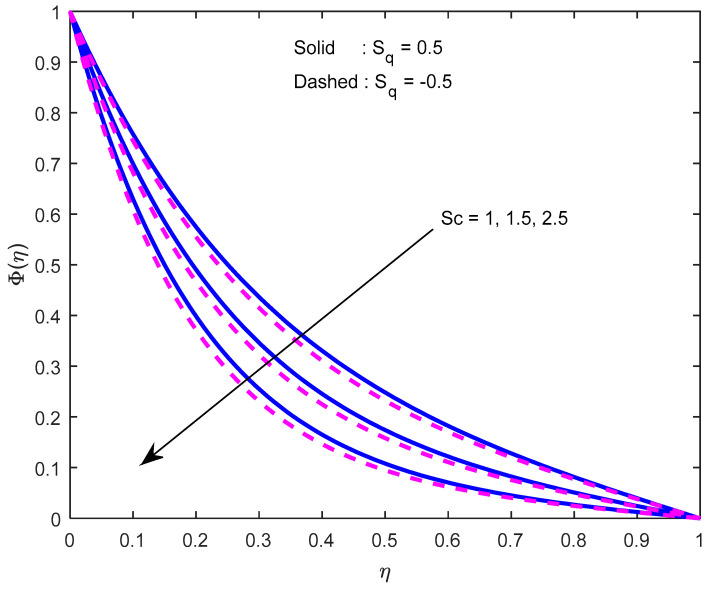
Effect of the Schmidt number on the concentration profile for various squeeze numbers.

**Figure 17:**
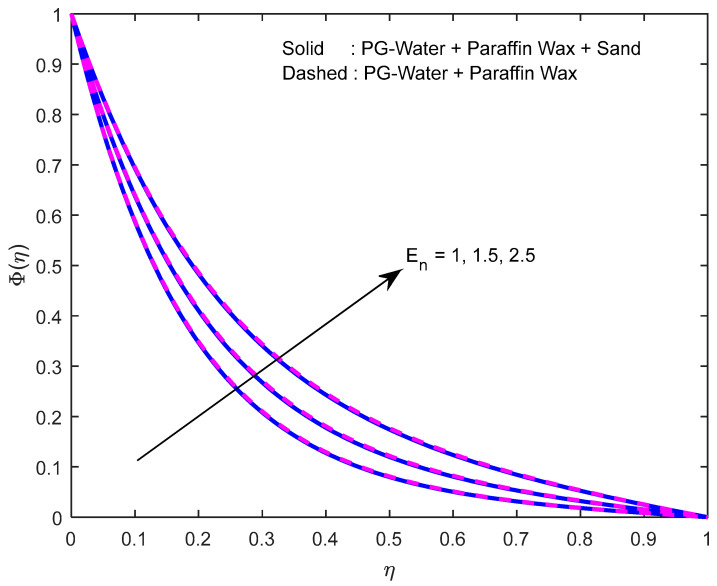
Effect of the activation energy parameter on the concentration profile.

**Figure 18:**
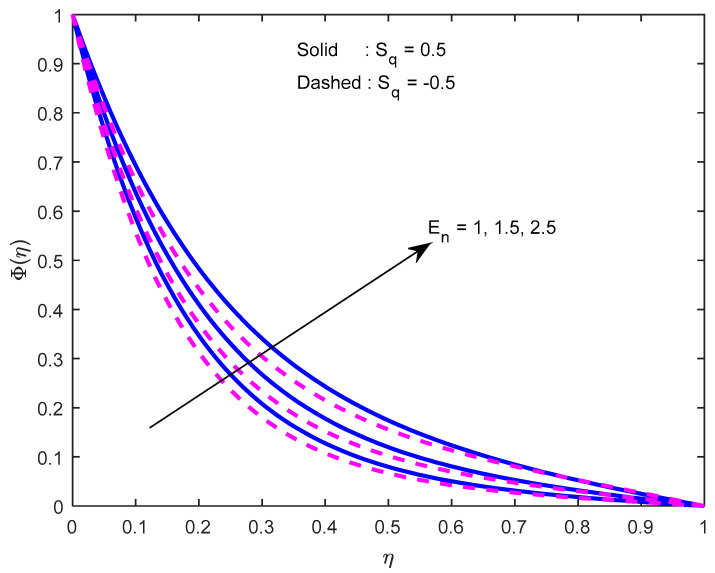
Effect of the activation energy parameter on the concentration profile for various squeeze numbers.

**Figure 19:**
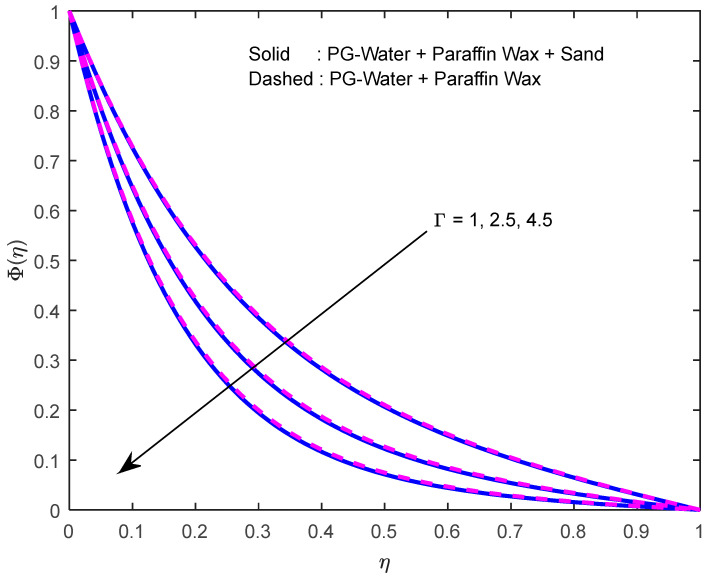
Effect of the reaction rate parameter on the concentration profile.

**Figure 20:**
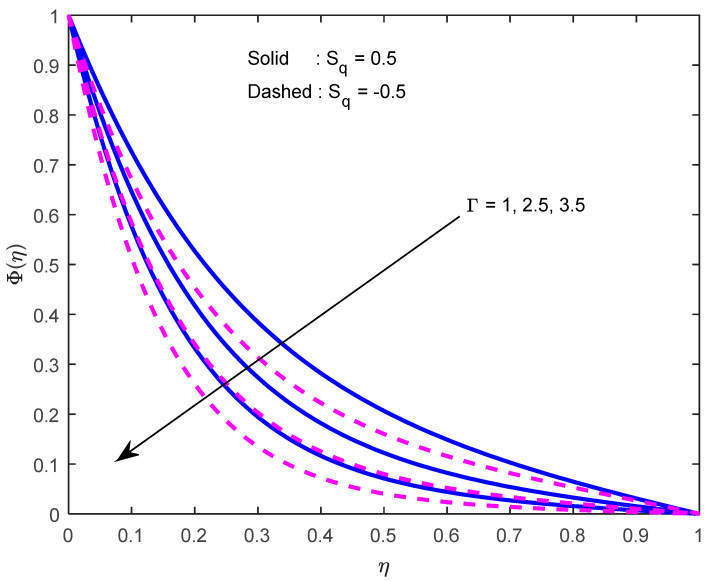
Effect of the reaction rate parameter on the concentration profile for various squeeze numbers.

**Figure 21:**
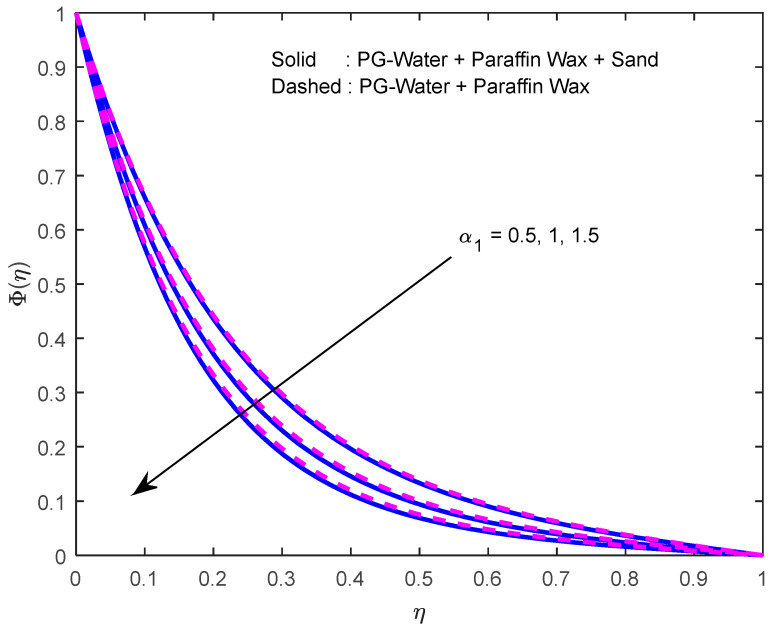
Effect of the temperature difference parameter on the concentration profile.

**Figure 22:**
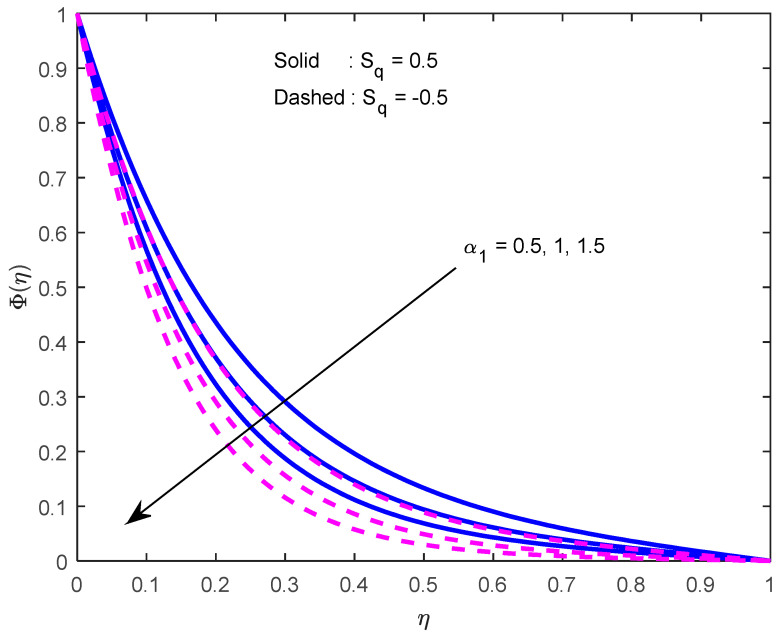
Effect of the temperature difference parameter on the concentration profile for various squeeze numbers.

Mass diffusivity minifies with a larger Schmidt number. So, concentration minifies with a larger Schmidt number (Figure 15 and Figure 16). Figure 17 and Figure 18 elucidate the impression of activation energy on the concentration profile. Generally, an increase in activation energy leads to a reduction in the threshold energy of the fluid, which, in turn, demonstrates the average kinetic energy. From the above condition, we can conclude that the average kinetic energy is less. Hence, diffusion will be less, which leads to a high concentration of the fluid. It is perceived that the fluid concentration is minified with a larger reaction rate parameter (Figure 19 and Figure 20). Higher temperature difference leads to lower molecular diffusivity. So, fluid concentration is minified with higher $\alpha_{1}$ (Figure 21 and Figure 22). We observe that profiles look higher in the case of a positive squeezing number contrasted with a negative squeezing number.

### 5.4. Statistical Analysis of Physical Parameters Using Correlation Coefficient

The correlation coefficient is a numerical measure of the association between two factors. The value of the coefficient lies between −1 (negative association) and 1 (positive association).

One can evaluate the correlation coefficient using:

$$\gamma =\frac{r\left(\sum yz\right)-\left(\sum y\right)\left(z\right)}{\sqrt{\left[r\sum y^{2}-{\left(\sum y\right)}^{2}\right]}\left[r\sum z^{2}-{\left(\sum z\right)}^{2}\right]}$$

Probable error (P.E) of the correlation coefficient helps with choosing the exactness and reliability of the coefficient value. The importance of the correlation relies upon the association between the coefficient value (γ) and P.E. If $\frac{\left|\gamma \right|}{P.E}>6$ (or |γ|>6P.E), then the correlation is significant, and if |γ|<P.E, then we say that the correlation is insignificant.

One can evaluate P.E by using $P.E=0.6745\frac{1-\gamma^{2}}{\sqrt{r}}$ where r is the number of observations.

We conducted a statistical analysis through a correlation coefficient to understand the impression of some important parameters on surface friction factor and transfer rates of heat and mass. Table 3 and Table 4 elucidate the relationship between surface drag force and some parameters (squeezing number, magnetic field, rotating parameter, and nanoparticle volume fraction parameter) near the lower and upper plates in two instances i.e., hybrid and mono nanofluids. It is witnessed that, except for the squeezing number, the other parameters (magnetic field, rotation parameter, and nanoparticle volumetric parameter) have a strong negative relationship with the friction factor at the lower plate. That means those three parameters deprecate the shear stress near the lower plate. Near the upper plate, it is acknowledged that the aforementioned parameters, except magnetic field, show reverse behavior on friction factor. Table 5 and Table 6 exhibit the relationship among N,$R_{a}$, and the Nusselt number near the lower and upper plates. We find a strong negative relationship between N and Nusselt number, and a strong positive relationship between $R_{a}$ and the heat transfer rate near the lower plate. This means that near the lower plate and larger heat source deprecates the heat transfer rate, and radiation parameter escalates the same. Near the upper plate, except for the radiation parameter, the heat source parameter displays a strong positive relationship with the heat transfer rate. Table 7 and Table 8 elucidate the relationship between $Sc,E_{n},\Gamma ,\alpha_{1}$, and the mass transfer rate near both plates. It is identified that the Sherwood number has a strong positive relationship with $Sc,\Gamma ,\alpha_{1}$, and a strong negative relationship with $E_{n}$, near the lower plate. This means that the Sherwood number minifies with larger $Sc,\Gamma ,\alpha_{1}$, and intensifies with larger $E_{n}$. It is noticed that the complete opposite relationship between $Sc,E_{n},\Gamma ,\alpha_{1}$ and the mass transfer rate occurs near the upper plate.

### 5.5. Entropy Generation and Bejan Number Profiles

**Figure 23:**
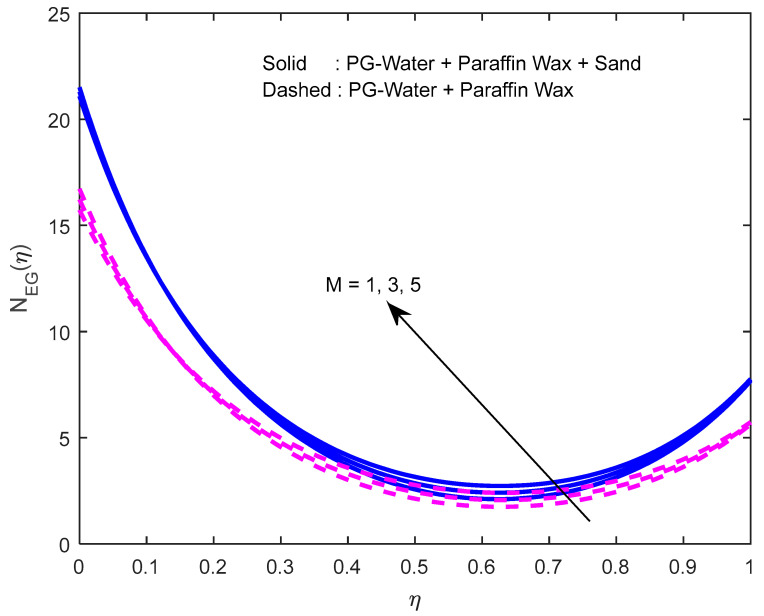
Effect of the magnetic field parameter on the entropy generation profile.

**Figure 24:**
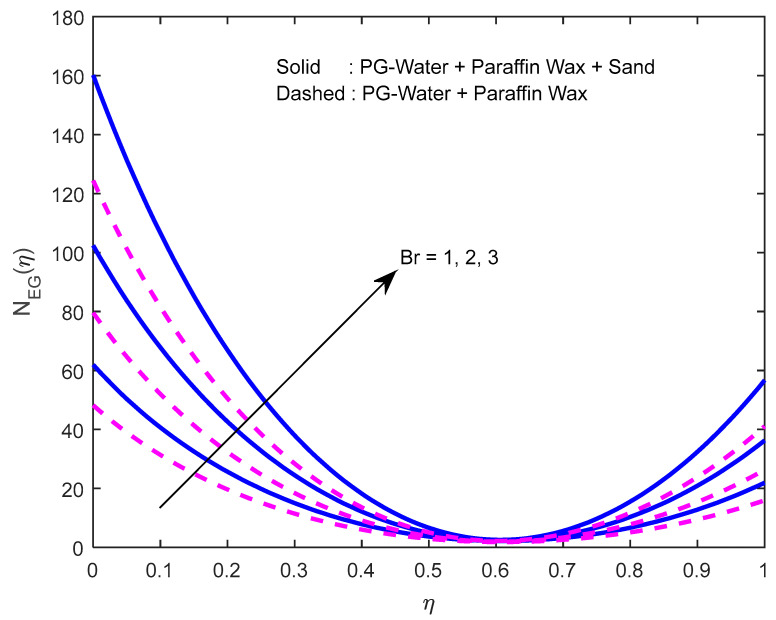
Effect of the Brinkmann number on the entropy generation profile.

**Figure 25:**
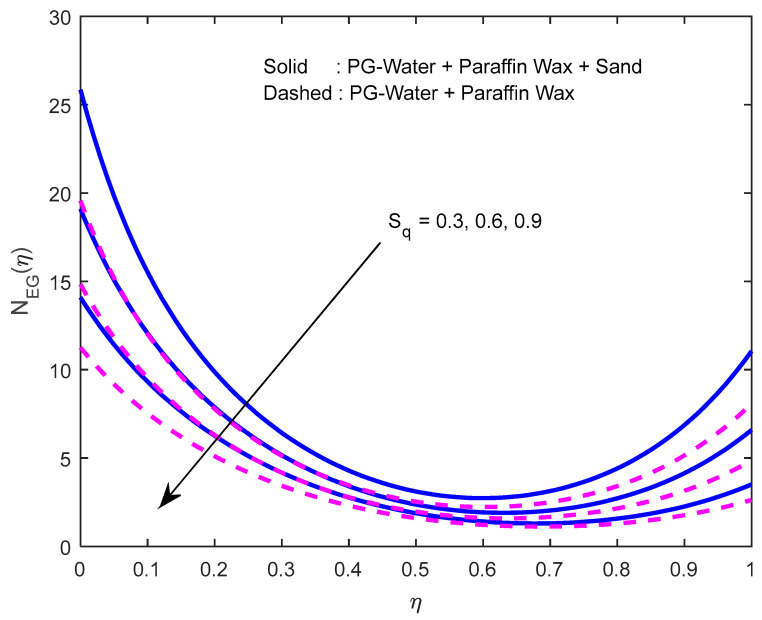
Effect of the squeeze number on the entropy generation profile.

**Figure 26:**
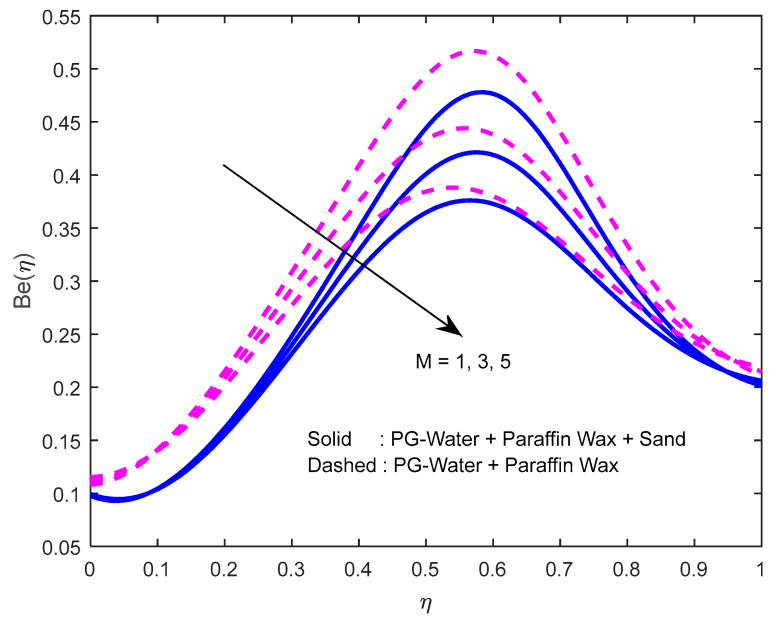
Effect of the magnetic field parameter on Bejan number profile.

**Figure 27:**
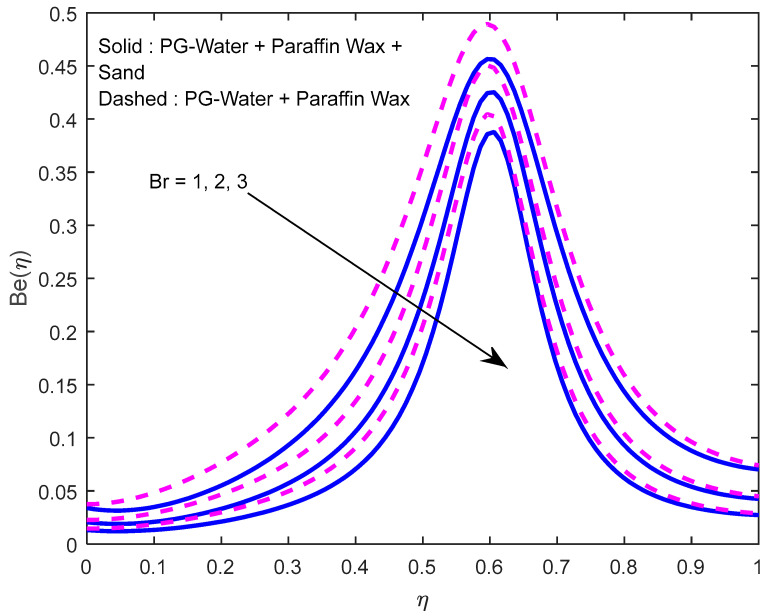
Effect of the Brinkmann number on Bejan number profile.

**Figure 28:**
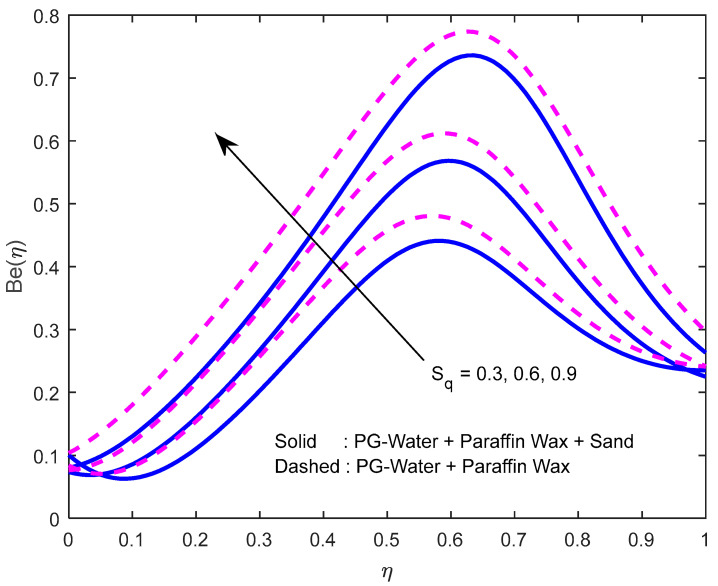
Effect of the squeeze number on Bejan number profile.

Since there is a direct relationship between entropy generation and the temperature of the fluid, we can deduce that M,Br are useful in enhancing entropy generation (Figure 23 and Figure 24). Figure 25 explicates the fact that the squeezing number minimizes entropy generation. We observe from Figure 26 and Figure 27 that the Bejan number minifies with larger M,Br (because, in each case, the irreversibility of the heat and mass transfer is eclipsed by the unchangeability of the other terms, including fluid friction). Figure 28 reveals that the squeezing number escalates the Bejan number.

## 6. Conclusions

A hybrid nanofluid, which is a combination of a propylene glycol–water mixture and paraffin wax–sand, may be utilized as a standby for a propylene glycol–water blend in the solar thermal systems. In addition, the squeezing nanofluid flow has applications in different fields, such as chemical engineering, the food industry, and polymer preparation. Therefore, this study investigates the squeezing flow of a propylene glycol and water mixture-based hybrid nanofluid between two parallel plates with activation energy and entropy generation. A shooting strategy is applied to unravel converted equations. Results and related parameters relationships are demonstrated in graphs and discussed. In addition, the study uses a statistical tool (correlation coefficient) to elucidate the impact of pertinent parameters on the concern parameters, such as the surface friction factor at both plates. Furthermore, the study results verify the available data from the literature, and show good agreement.

The primary conclusions of the current investigation are listed below:

- A larger squeezing number intensifies velocity profiles;
- A raise in the rotation parameter deprecates the normal velocity (in the x-direction) in the lower half, and ameliorates it the same in the upper half;
- An escalation in fluid temperature is recognized with larger N;
- Fluid concentration reduces with higher reaction rate parameters, and raises with melioration in activation energy;
- The magnetic field, rotation parameter, and nanoparticle volumetric parameter have a strong negative relationship with the friction factor at the lower plate;
- The squeezing number escalates the friction factor near the lower plate, and depreciates it near the upper plate. N has a strong negative relationship with the heat transfer rate near the lower plate, and a strong positive correlation with the same phenomena near the upper plate;
- The Sherwood number minifies with larger $Sc,\Gamma ,\alpha_{1}$, and intensifies with larger $E_{n}$ near the lower plate.

## Figures and Tables

**Table 2:** Verification of present results with earlier outcomes under special conditions.

		Munawar et al. [3]		Current Study			
				Shooting Method		Bvp4c Method	
S	$S_{q}$	$f^{\prime \prime }\left(1\right)$	$g^{\prime }\left(1\right)$	$f^{\prime \prime }\left(1\right)$	$g^{\prime }\left(1\right)$	$f^{\prime \prime }\left(1\right)$	$g^{\prime }\left(1\right)$
0	2	−4.5827056	−1.2304429	−4.582984	−1.230411	−4.582984	−1.230411
0.3		−2.6227888	−0.8059641	−2.622739	−0.805942	−2.622739	−0.805942
0.6		−0.7312990	−0.3611836	−0.731299	−0.361177	−0.731299	−0.361177
0.9		1.0924124	0.1059088	1.092497	0.105892	1.092497	0.105892
1.2		2.8491655	0.5975976	2.849166	0.597586	2.849166	0.597586
0.5	−1	7.7031724	3.0250739	7.703171	3.025059	7.703171	3.025059
	0	4.8235909	1.4031897	4.823590	1.403201	4.823590	1.403201
	1	1.8091719	0.2966841	1.809170	0.296684	1.809170	0.296684
	2	−1.3542292	−0.5118183	−1.354199	−0.511811	−1.354199	−0.511811
	3	−4.6700503	−1.1328529	−4.670051	−1.132848	−4.670051	−1.132848

**Table 3:** Correlation coefficient (γ) and probable error (P.E) for surface drag force at the lower plate.

	$C_{f_{0}}$					
	Hybrid Nanofluid			Nanofluid		
	γ	P.E	$\frac{\left|\gamma \right|}{P.E}$	γ	P.E	$\frac{\left|\gamma \right|}{P.E}$
M	−0.9999	0.000044	22725	−0.9999	0.000044	22725
$S_{q}$	0.9870	0.005630	175.31	0.9792	0.008990	108.92
Ω	−0.9661	0.014555	66.38	−0.9666	0.014337	67.42
$\phi_{1}$	−0.9296	0.029634	31.37	−0.9281	0.030245	30.69

**Table 4:** Correlation coefficient (γ) and probable error (P.E) for surface drag force at the upper plate.

	$C_{f_{1}}$					
	Hybrid Nanofluid			Nanofluid		
	γ	P.E	$\frac{\left|\gamma \right|}{P.E}$	γ	P.E	$\frac{\left|\gamma \right|}{P.E}$
M	−0.9953	0.002051	485.28	−0.9725	0.011828	82.22
$S_{q}$	−0.9999	0.000044	22725	−0.9999	0.000044	22725
Ω	0.9655	0.014795	65.26	0.9655	0.014795	65.26
$\phi_{1}$	0.9316	0.028827	32.32	0.9316	0.028827	32.32

**Table 5:** Correlation coefficient (γ) and probable error (P.E) for local Nusselt number at the lower plate.

	$Nu_{0}$					
	Hybrid Nanofluid			Nanofluid		
	γ	P.E	$\frac{\left|\gamma \right|}{P.E}$	γ	P.E	$\frac{\left|\gamma \right|}{P.E}$
N	−0.9999	0.000044	22725	−0.9999	0.000044	22725
$R_{a}$	0.9999	0.000044	22725	0.9999	0.000044	22725

**Table 6:** Correlation coefficient (γ) and probable error (P.E) for local Nusselt number at the upper plate.

	$Nu_{1}$					
	Hybrid Nanofluid			Nanofluid		
	γ	P.E	$\frac{\left|\gamma \right|}{P.E}$	γ	P.E	$\frac{\left|\gamma \right|}{P.E}$
N	0.9996	0.000174	5744.83	0.9996	0.000174	5744.83
$R_{a}$	0.9999	0.000044	22725	0.9999	0.000044	22725

**Table 7:** Correlation coefficient (γ) and probable error (P.E) for local Sherwood number at the lower plate.

	$Sh_{0}$					
	Hybrid Nanofluid			Nanofluid		
	γ	P.E	$\frac{\left|\gamma \right|}{P.E}$	γ	P.E	$\frac{\left|\gamma \right|}{P.E}$
Sc	0.9997	0.000130	7690	0.9997	0.000130	7690
$E_{n}$	−0.9832	0.007267	135.3	−0.9831	0.007310	134.49
Γ	0.9999	0.000044	22725	0.9999	0.000044	22725
$\alpha_{1}$	0.9989	0.000480	2081.04	0.9989	0.000480	2081.04

**Table 8:** Correlation coefficient (γ) and probable error (P.E) for local Sherwood number at the upper plate.

	$Sh_{1}$					
	Hybrid Nanofluid			Nanofluid		
	γ	P.E	$\frac{\left|\gamma \right|}{P.E}$	γ	P.E	$\frac{\left|\gamma \right|}{P.E}$
Sc	−0.9824	0.007616	128.99	−0.9839	0.006983	140.9
$E_{n}$	0.9954	0.002007	495.96	0.9947	0.002313	430.05
Γ	−0.9999	0.000044	22725	−0.9999	0.000044	22725
$\alpha_{1}$	−0.9975	0.001091	914.3	−0.9981	0.000829	1203.98

## Data Availability

The manuscript has no associated data.

## Nomenclature

**Nomenclature**		**Greek Symbols**	
B(t)	Magnetic field (Tesla)	$\alpha_{1}$	Temperature difference parameter
Be	Bejan number	$\alpha_{2}$	Concentration ratio parameter
Br	Brinkman number	Γ	Reaction rate parameter
C	Hybrid nanofluid concentration	γ	Characteristic parameter with dimension of time^−1^
$C_{p}$	Specific heat (J/kg K)	η	Similarity variable
$D_{m}$	Molecular diffusivity	θ	Dimensionless temperature
$E_{a}$	Activation energy	*μ*	Dynamic viscosity (kg/m·s)
$E_{n}$	Activation energy parameter	υ	Kinematic viscosity (m^2^/s)
f	Dimensionless axial velocity	ρ	Density (kg/m^3^)
g	Dimensionless transverse velocity	$\rho C_{p}$	Fluid heat capacity (J/Km^3^)
H*	Diffusion parameter	σ	Electrical conductivity (S/m)
k	Thermal conductivity (W/m K)	σ*	Stefan–Boltzmann constant (W/m^2^K^4^)
k*	Mean absorption coefficient (1/m)	Φ	Dimensionless nanoparticle concentration
$k_{1}$	Boltzmann constant (J/K)	ϕ	Nanoparticle volume fractions
$k_{r}$	Chemical reaction rate (1/s)	Ω	Rotation parameter
M	Magnetic field parameter	Ω*	Angular velocity (m^2^/s)
N	Heat source parameter	ω	Rotational velocity (1/s)
n	Fitted rate constant		
$N_{EG}$	Entropy generation		
$Nu_{x}$	Nusselt number	**Subscripts**	
p	Pressure	f	Fluid
Pr	Prandtl number	nf	Mono nanofluid
$q_{w}$	Wall heat flux	hnf	Hybrid nanofluid
$Q_{0}$	Dimensional heat source parameter (W/m^3^K)	s	Nanomaterials
$\bar{R}$	Universal gas constant		
$R_{a}$	Radiation parameter		
$V_{0}$	Permeability parameter		
S	Suction parameter		
$s_{w}$	Wall mass flux		
Sc	Schmidt number		
$S_{q}$	Squeeze number		
T	Fluid temperature (K)		
u, v, w	Velocity component along x, y, and z-directions (m/s)		
$V_{h}$	Squeezing velocity (m/s)		
a	Stretching rate of lower plate		
